# Platelet function testing in atrial fibrillation patients undergoing percutaneous coronary intervention

**DOI:** 10.1007/s11239-022-02723-4

**Published:** 2022-11-12

**Authors:** Ioannis Lianos, Charalampos Varlamos, Despoina-Rafailia Benetou, Christos Mantis, Konstantinos Kintis, Vassiliki-Maria Dragona, Ioannis Kanakakis, Dimitrios Sionis, Sotirios Patsilinakos, Dimitrios Alexopoulos

**Affiliations:** 1 2nd Department of Cardiology, Attikon University Hospital, National and Kapodistrian University of Athens Medical School, Rimini 1, Chaidari, 12462 Athens, Greece; 2 Department of Cardiology, Konstantopoulion Hospital, Athens, Greece; 3 3rd Department of Internal Medicine, Red Cross Hospital, Athens, Greece; 4 Department of Clinical Therapeutics, “Alexandra” University Hospital, Athens, Greece; 5 Department of Cardiology, Sismanoglion Hospital, Athens, Greece

**Keywords:** P2Y_12_ inhibitor, Double antithrombotic therapy, Platelet function, Atrial fibrillation, Percutaneous coronary intervention

## Abstract

Platelet function testing (PFT) could be a useful clinical tool to guide individualized antithrombotic treatment in patients with atrial fibrillation (AF) undergoing percutaneous coronary intervention (PCI). We aimed to investigate platelet reactivity (PR) in the context of a contemporary registry. “Real-world” data were retrieved from a nationwide, multicenter, observational study of AF patients on oral anticoagulants (OAC) undergoing PCI. Patients treated with a P2Y_12_ inhibitor, namely clopidogrel or ticagrelor, as part of double or triple antithrombotic therapy, were submitted to PFT before discharge and were followed up for 12 months. Out of 101 patients included in the study, 66 were submitted to PFT while on clopidogrel and 35 while on ticagrelor; PR was 162.9 ± 68 PRU and 46.02 ± 46 PRU, respectively (P < 0.001). High on-treatment PR (HTPR) was observed in 15 patients under clopidogrel (22.7%); 7 of them escalated to ticagrelor. Low on-treatment PR (LTPR) was found in 9 clopidogrel and 28 ticagrelor-treated patients (13.6% vs. 80%, P < 0.001), of whom only 1 de-escalated to clopidogrel. PR did not differ by OAC regimen. PFT results had no impact on aspirin prescription at discharge, while failed to predict significant bleeding events at follow up. Ticagrelor administration led to lower PR and lower incidence of HTPR in comparison with clopidogrel. Physicians’ behavior in response to knowledge of a patient’s PR was variable. Further studies are required to elucidate the role of PFT as a tool to guide individualized antithrombotic treatment in this clinical scenario.

**Supplementary Information:**

The online version contains supplementary material available at 10.1007/s11239-022-02723-4.

## Introduction

Antithrombotic treatment in atrial fibrillation (AF) patients with an indication for oral anticoagulation (OAC) undergoing percutaneous coronary intervention (PCI) poses a significant therapeutic dilemma. On the one hand, this subgroup of patients has an increased ischemic risk, including both stroke and stent thrombosis (ST) [1], while, on the other hand, the “traditional” approach of triple antithrombotic therapy (TAT) consisting of dual antiplatelet therapy (DAPT) with aspirin and a P2Y_12_ inhibitor plus OAC is associated with high bleeding rates [2]. Recent randomized controlled trials (RCTs) compared dual antithrombotic therapy (DAT), with a non-vitamin K oral anticoagulant (NOAC) plus single antiplatelet therapy (SAPT), to TAT, with vitamin K antagonist (VKA) plus DAPT, and make a compelling case regarding safety of DAT with an antiplatelet agent, mainly clopidogrel, when compared with TAT [3–6]. However, there are concerns in case of aspirin omission, that patients on solely clopidogrel treatment might be exposed in increased risk of ST [1, 7]. Among clopidogrel-treated patients, high platelet reactivity (PR) is commonly encountered, with incidence varying from 16 to 50% [8, 9]. Τhere is paucity of data concerning the incidence of high on-treatment platelet reactivity (HTPR) in AF patients treated with NOACs undergoing PCI [10]. In spite of HTPR risk, clopidogrel is the preferred agent in this clinical context, with the novel P2Y_12_ inhibitors, ticagrelor and prasugrel, underrepresented in the aforementioned trials [3–6, 11]. In this scenario, platelet function testing (PFT) might serve as a clinical tool to guide tailoring of antithrombotic regimen, i.e. intensification of treatment via escalation to a more potent agent or aspirin addition in case of initial DAT selection. Furthermore, PFT has been suggested as a useful tool to identify patients with an increased risk of bleeding [12, 13].

In this background, this study aims to investigate PR in AF patients on OAC undergoing PCI, in the context of a real-life contemporary registry, and its potential impact on antithrombotic agent choices and patient outcomes.

## Methods

Greek AntiPlatElet Atrial Fibrillation (GRAPE-AF) registry is a nationwide, prospective, single-arm, observational, non-interventional, cohort study, enrolling patients with non-valvular AF on OAC undergoing PCI with stent implantation in 18 PCI centers in Greece. The study, with rationale and design previously described in detail, aims to provide “real world” data regarding antithrombotic therapy [14]. Overall, 653 patients have been recruited (exclusion criteria in supplementary Table 1). A written informed consent was obtained from all participants. The study complies with the declaration of Helsinki and was approved by the Ethic Committee of each participating hospital. GRAPE-AF study is registered at ClinicalTrials.gov: NCT03362788.

Between March 2018 and October 2019, in the context of GRAPE-AF registry, we assessed PR in anticoagulated AF patients undergoing PCI in 6 hospitals capable of performing PFT using the VerifyNow assay (Accriva Diagnostics, San Diego, CA). Eligible patients, treated with a P2Y_12_ inhibitor, namely clopidogrel or ticagrelor (per treating physicians’ choice), as part of DAT with OAC or TAT with OAC plus aspirin, were submitted in PFT pre-discharge, after index PCI. Results were available to the attending physician before patient’s discharge and used upon his discretion.

For PR assessment, peripheral venous blood samples were drawn in 2 ml Greiner Bio-One Vacuette® partial fill blood collection tubes containing 3.2% sodium citrate, 2–4 h after P2Y_12_ inhibitor maintenance dose and at least 12 h post – loading dose (600 mg of clopidogrel or 180 mg of ticagrelor) in case of P2Y_12_ inhibitor naive patients. PR was measured via the point of care P2Y_12_ VerifyNow platelet function assay by an investigator blinded to the antithrombotic regimen. Results were reported in P2Y_12_ reaction units (PRU) and percent inhibition, which was calculated as: ([BASE - PRU]/ BASE) × 100. A PRU > 208 and a PRU < 85 were considered as HTPR and low on-treatment platelet reactivity (LTPR), respectively [8].

Follow-up visits were performed through telephone or personal contact in 1, 6 and 12 months after enrollment. Collected data included major adverse cardiovascular and cerebrovascular events (MACCE), bleeding complications and side effects of antithrombotic therapy, as well as information regarding patients’ adhesion or changes to the prescribed treatment regimen.

The prespecified primary endpoints used for sample size calculation of this sub-study was PR level and HTPR rate between patients treated with clopidogrel vs. ticagrelor. Secondary endpoints included bleeding (per the Bleeding Academic Research Consortium [BARC] definition) and major adverse cardiovascular events (MACE), i.e. cardiovascular death, myocardial infarction, stroke, stent thrombosis or unplanned coronary revascularization rates, during the first year post index PCI.

Continuous data are presented as means ± standard deviation (SD) and compared using the Student’s t-test whereas categorical variables are presented as frequencies and group percentages and compared using the chi-square or Fisher exact test. Kaplan-Meier curves for bleeding-free survival were obtained for each P2Y_12_ inhibitor subgroup and compared using the log-rank test. If multiple bleeds were recorded for a patient, categorization was based on the first bleeding event at the highest level of severity experienced. All tests were two-tailed, and statistical significance was considered for P values < 0.05.

Approximately 600 patients during 1 year were initially planned to participate in GRAPE-AF registry. Out of them, 70% and 30% were predicted to receive clopidogrel and ticagrelor respectively, as part of their antithrombotic therapy. Taking into account that PR assessment wouldn’t be available in all participating centres, we planned to assess PR in 120 patients. Out of them 36 and 84 were expected to receive ticagrelor and clopidogrel respectively. Assuming a PR of 35 ± 30 PRU and 100 ± 70 PRU for ticagrelor and clopidogrel respectively, group sample sizes of 36 and 84 would achieve > 95% power to detect a difference of at least 65.0 PRU between groups, with estimated SD of 35.0 and 70.0 respectively and with a significance level (alpha) of 0.05, assuming that the actual distribution is not normal [14]. When assuming an HTPR rate of 3% and 25% in ticagrelor and clopidogrel group respectively, the same group sample sizes were predicted to achieve 88% power to detect an absolute difference between the group proportions of 22% with a two-sided Fisher’s exact test, at a 0.05 significance level.

Analyses were performed using SPSS (version 22.0; SPSS Inc., Chicago, IL, USA) and GraphPad Prism (version 5; GraphPad Software, Inc. USA) software.

## Results

Overall, 653 patients have been enrolled in the GRAPE-AF registry. Out of these, a total of 101 patients with PFT were included in the present sub-study. Table 1 presents characteristics of study population per P2Y_12_ inhibitor group at PFT with 66 and 35 patients treated with clopidogrel and ticagrelor, respectively. All patients received periprocedurally aspirin and till discharge. There was a tendency for more common use of ticagrelor in acute coronary syndrome than in stable coronary artery disease patients and ticagrelor use in the form of DAT (without aspirin at discharge). At PFT, VKA were administered to 13 patients (12.9%), while 88 (87.1%) received NOAC; 55 patients received rivaroxaban, 23 dabigatran and 10 apixaban (Suppl. Figure S1).

**Table 1:** Patients’ characteristics per P2Y12 inhibitor at platelet function testing assessment

Patients’ characteristics	Total population (N = 101)	Clopidogrel (n = 66)	Ticagrelor (n = 35)	P-value
Male sex	81 (80%)	54 (82%)	27 (77%)	0.575
Age (SD)	70.8 (10.7)	73.2 (9.1)	66.2 (12)	0.001
BMI (SD)	29 (5.6)	28.9 (4.4)	29.2 (7.5)	0.797
Smoking	29 (28.7%)	12 (18.2%)	17 (48.6%)	0.001
Hypertension	81 (80%)	52 (79%)	29 (83%)	0.625
Diabetes	39 (38%)	25 (38%)	14 (40%)	0.835
Dyslipidemia	63 (62%)	44 (67%)	19 (54%)	0.222
Previous myocardial infarction	33 (32%)	19 (29%)	14 (40%)	0.253
Previous percutaneous coronary intervention	26 (25%)	17 (26%)	9 (26%)	0.996
Previous coronary-artery by-pass grafting	14 (14%)	10 (15%)	4 (11%)	0.766
Previous stent thrombosis	4 (4%)	1 (2%)	3 (9%)	0.119
Renal failure (30 ml/min/1.73m2 < eGFR < 60ml/min/1.73m2)	9 (9%)	6 (9%)	3 (9%)	1.000
Index event				0.067
STEMI	20 (21%)	9 (14%)	11 (31%)	
NSTEMI	19 (19%)	11 (17%)	8 (23%)	
Unstable Angina	28 (27%)	19 (29%)	9 (26%)	
Stable coronary artery disease	34 (33%)	27 (41%)	7 (20%)	
Aspirin at discharge	40 (40%)	30 (45%)	10 (29%)	0.099
Radial access	86 (85%)	53 (80%)	33 (94%)	0.060
Number of lesions treated				
1	62 (62%)	41 (62%)	21 (60%)	0.835
2	31 (30%)	20 (30%)	11 (31%)	0.907
≥ 3 lesions treated	8 (8%)	5 (8%)	3 (9%)	1.000
Drug-Eluting Stent	100 (99%)	65 (98%)	35 (100%)	1.000
≥ 3 stents implanted	18 (18%)	11 (17%)	7 (20%)	0.677
Average stent length (SD)	35.8 (21.9)	32.7 (20.0)	41.4 (24.4)	0.060
Total stent length ≥ 60 mm	14 (14%)	8 (12%)	6 (17%)	0.550
Bifurcation	8 (8%)	6 (9%)	2 (6%)	0.711

**Fig. 1:**
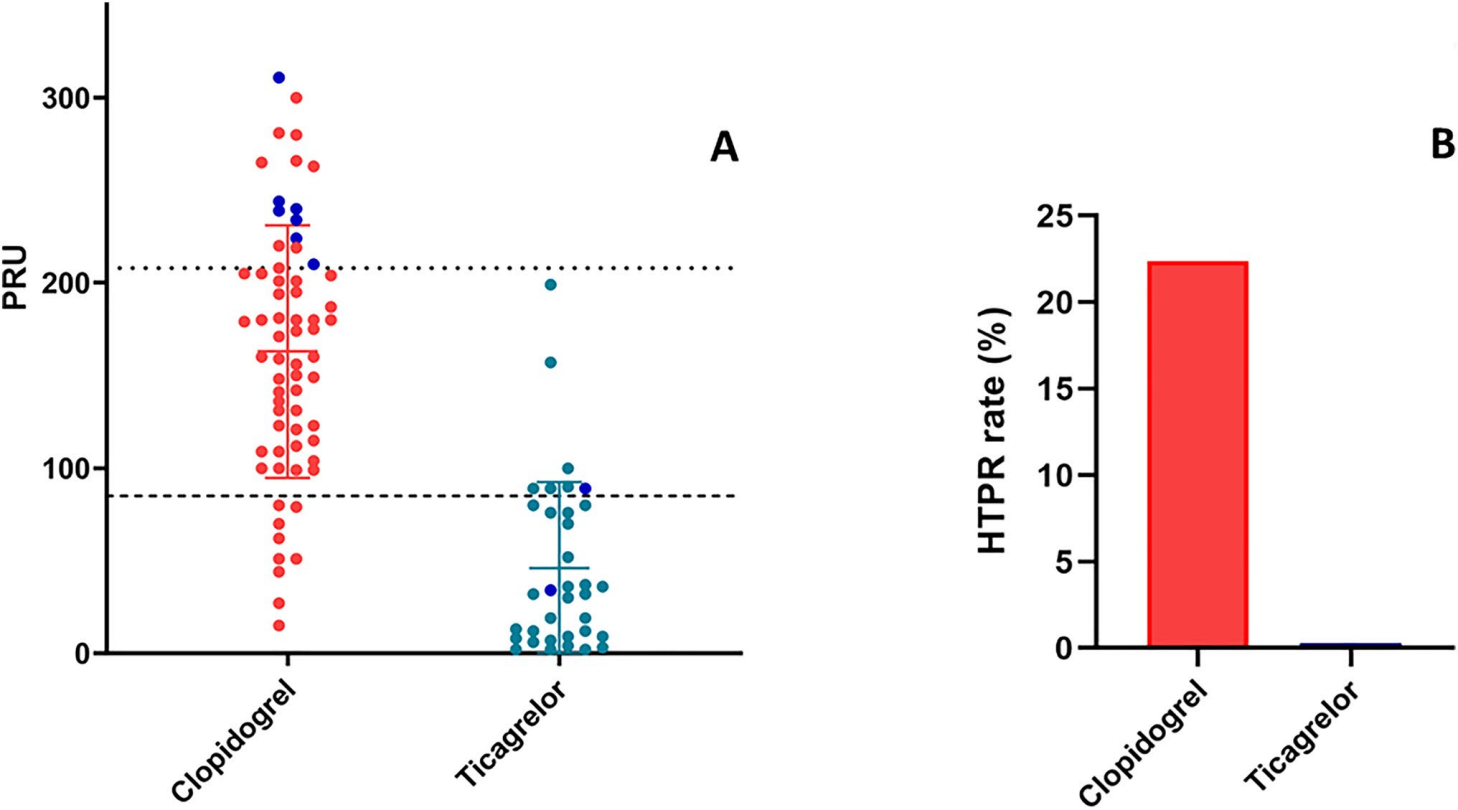
Individual platelet reactivity values (**A**) and HTPR rate (**B**) by P2Y12 inhibitor. ^...^HTPR threshold; ---LTPR thresold. * PRU* platelet reaction units; * HTPR* high on treatment platelet reactivity

Mean PRU values were 162.91 ± 68 for patients on clopidogrel and 46.03 ± 46 for patients on ticagrelor (mean treatment difference = 116.88, 95% confidence interval (CI) = 94.05 to 139.71, P < 0.001). HTPR was observed in 15 patients receiving clopidogrel (22.7%), while in none of ticagrelor arm (P = 0.001). Individual PRU values and HTPR rates according to antiplatelet agent are shown in Fig. 1. In contrast, LTPR was found in 9 patients receiving clopidogrel (13.6%) and in 28 (80%) ticagrelor-treated patients, (P < 0.0001). The percentage of platelet inhibition was lower among clopidogrel compared to ticagrelor-treated patients (26.56 ± 25 vs. 79.57 ± 19; mean treatment difference = − 53.01, 95% confidence interval (CI) = − 61.80 to − 44.23, P < 0.0001).

**Fig. 2:**
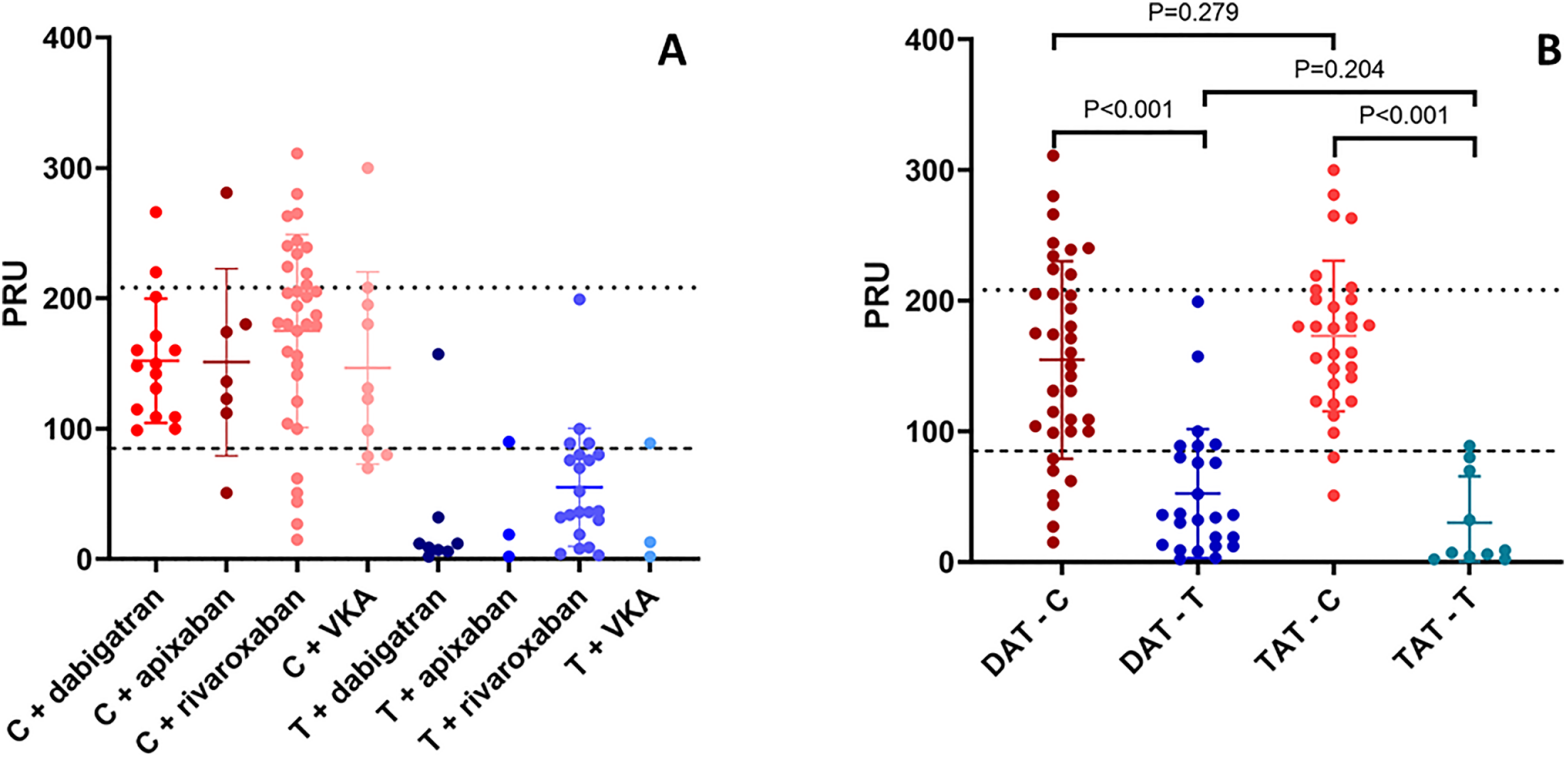
Individual PRU values by (**A**) antiplatelet and anticoagulant agent administered and (**B**) DAT vs. TAT stratified by P2Y12 inhibitor. Bars represent mean PRU value and whiskers represent SD. HTPR threshold; * LTPR threshold*; *C*, clopidogrel; *DAT* double antithrombotic therapy; *PRU* platelet reaction units; *TAT*TAT triple antithrombotic therapy; *T* ticagrelor; *VKA* vitamin K antagonist

PR was similar between patients receiving different anticoagulant agents in their regimen: 130 ± 87 for rivaroxaban, 110 ± 77 for dabigatran, 117 ± 83 for apixaban and 121 ± 83 for VKA; P = 0.810. PR values by antiplatelet and anticoagulant agent administered are shown in Fig. 2 A. Among the 101 study patients, PR did not differ significantly between patients receiving DAT or TAT (mean PRU 112.69 ± 83 vs. 137.23 ± 82; mean treatment difference = − 24.54, 95% confidence interval (CI) = − 57.83 to 8.75, P = 0.147). No difference in PR was observed in data analysis stratified by type of antiplatelet agent (DAT vs. TAT on clopidogrel, mean PRU 154.56 ± 76 vs. 172.93 ± 58, mean treatment difference = − 18.38, 95% confidence interval (CI) = − 51.17 to 14.42, P = 0.267; DAT vs. TAT on ticagrelor, mean PRU 52.40 ± 49 vs. 30.10 ± 36, mean treatment difference = 22.30, 95% confidence interval (CI) = − 12.75 to 57.35, P = 0.204). Among DAT receiving patients, PR was lower in ticagrelor than in clopidogrel treated patients. Similarly, among TAT receiving patients, PR was lower in ticagrelor than in clopidogrel treated patients, Fig. 2B.

**Fig. 3:**
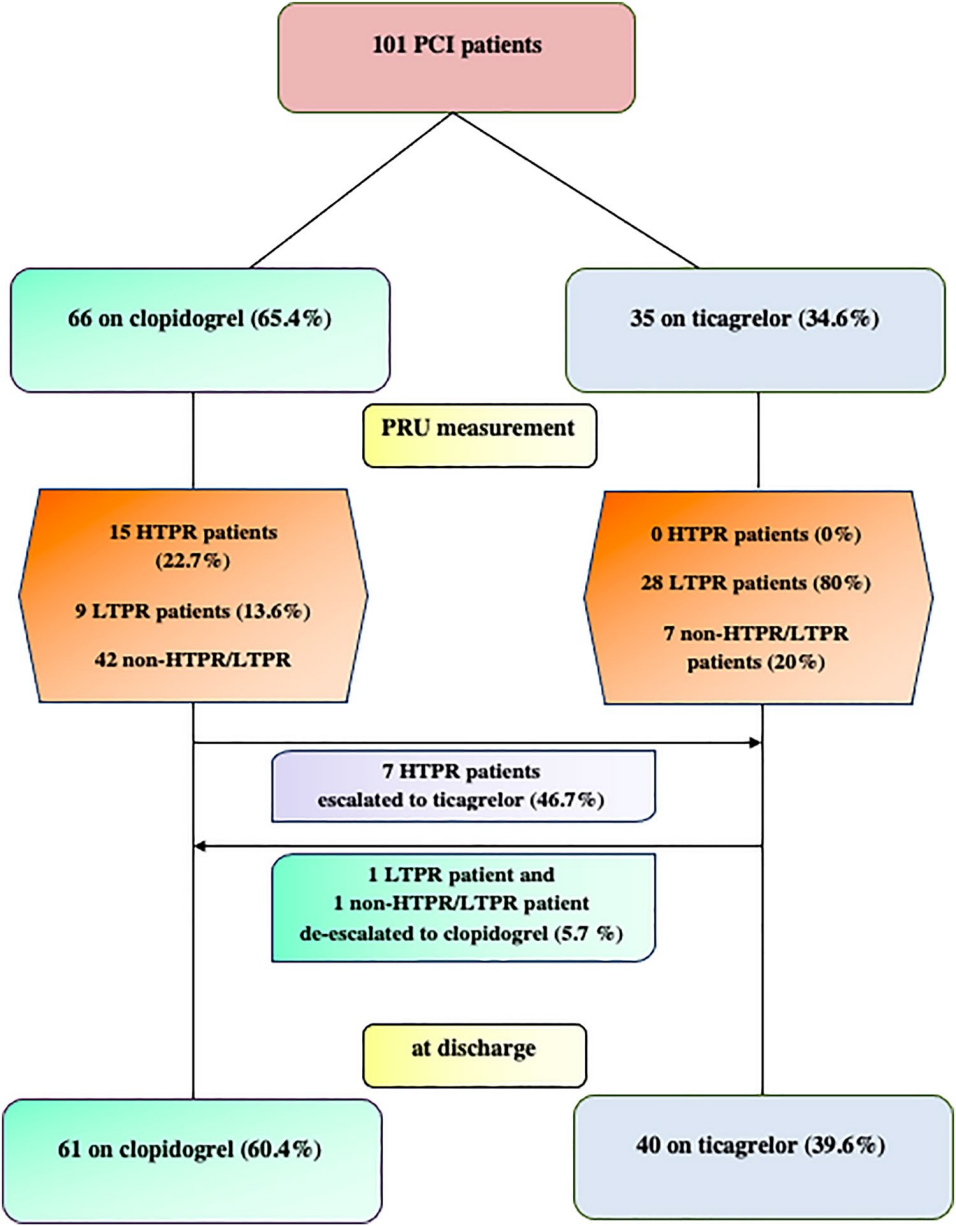
Study's patient flow chart according to platelet function testing results and subsequent antiplatelet treatment *HTPR* high on treatment platelet reactivity; *LTPR* low on treatment platelet reactivity;* PCI* percutaneous coronary intervention;* PRU* platelet reaction units

Study’s patient flow chart according to PFT and subsequent antiplatelet treatment is presented in Fig. 3. Out of 15 HTPR patients, 7 (47%) had their treatment escalated to ticagrelor (6 on DAT with OAC and 1 on TAT with OAC plus aspirin), 5 patients (33%) received aspirin on top of clopidogrel plus OAC and only 3 patients (20%) remained on clopidogrel plus OAC. Out of 28 LTPR patients on ticagrelor, only 1 de-escalated to clopidogrel (on DAT). TAT and DAT was prescribed in 6 (40%) and 9 (60%) HTPR patients, respectively, with similar rates compared to the non-HTPR population: 34/86 (39.5%) and 52/86 (60.5%) discharged on TAT and DAT, respectively (P = 0.973). TAT and DAT was prescribed in 11 (29.7%) and 26 (70.3%) LTPR patients, respectively, with rates also not significantly different compared to the non-LTPR population: 29/64 (45.3%) and 35/64 (54.7%) discharged on TAT and DAT, respectively (P = 0.183). Overall, 40 patients (39.6%) received ticagrelor while 61 patients (60.4%) received clopidogrel at discharge. Moreover, 40 patients (39.6%) received aspirin as part of TAT -mostly (24/40) for a pre-specified duration of 1 month- while 61 patients (60.4%) received DAT at discharge.

During the 12-month follow up, bleeding events occurred in 37 out of 101 patients (36.6%) overall, though BARC Type ≥ 2 in 16 (15.8%) (Table 2). Regarding antithrombotic regimen at discharge, bleedings were recorded in 13/40 patients on TAT (32.5%) and 24/61 (39.3%) patients on DAT. At the time of bleeding occurrence, 7 patients were under TAT and 30 patients were under DAT. A significant number of bleedings (27%) occurred within the first month of follow-up. Focusing on antiplatelet agent, 23 out of 61 clopidogrel-treated patients (37.7%) and 14 out of 40 ticagrelor-treated patients (35%) suffered from a bleeding event (P = 0.78). Clinically significant bleeding (BARC ≥ 2) was numerically more common, though not statistically significant in ticagrelor vs. clopidogrel treated patients (22.5% vs. 11.4%, P = 0.14). Clinically significant bleeding event-free survival during 12-month follow-up period is shown in Suppl. Figure 2. Of note, the majority of bleedings observed in ticagrelor arm were BARC ≥ 2 in contrast to clopidogrel arm (9/14 bleedings in ticagrelor arm vs. 7/23 bleedings in clopidogrel arm, P = 0.044).

**Table 2:** One year bleeding events rates by BARC type according to antithrombotic therapy at discharge

Bleeding BARC	Total (N = 101)	Clopidogrel (n = 61)	Ticagrelor (n = 40)	Aspirin (n = 40)	No Aspirin (n = 61)	NOAC (n = 88)	VKA (n = 13)
Type 1	21 (20.8%)	16 (26.2%)	5 (12.5%)	5 (12.5%)	16 (26.2%)	18 (20%)	3 (23%)
Type 2	9 (8.9%)	4 (6.5%)	5 (12.5%)	5 (12.5%)	4 (6.6%)	9 (10.2%)	0 (0%)
Type 3a	3 (2.9%)	1 (1.6%)	2 (5%)	2 (5%)	1 (1.6%)	3 (3.4%)	1 (7.7%)
Type 3b	4 (3.9%)	2 (3.2%)	2 (5%)	1 (2.5%)	3 (4.9%)	3 (3.4%)	0 (0%)
Type ≥ 2	16 (15.8%)	7 (11.5%)	9 (22.5%)	8 (20%)	8 (13.1%)	15 (17%)	1 (7.6%)
Total	37 (36.6%)	23 (37.7%)	14 (35%)	13 (32.5%)	24 (39.3%)	33 (37.5%)	4 (30.8%)

*BARC Bleeding* Academic Research Consortium, *NOAC novel* oral Anticoagulants, *VKA vitamin-K* antagonists

In patients suffering from any bleeding, PR at PFT was 119 ± 83 PRU compared to 124 ± 83 PRU in those who did not (P = 0.748). Mean PR in patients with BARC ≥ 2 bleeding events was 105 ± 96 PRU compared to 126 ± 80 PRU in patients with minor or no events (P = 0.370).

Regarding LTPR patients, bleeding events occurred in 16 out of 37 (43.2%) compared to 21 out of 64 (32.8%) non-LTPR patients (P = 0.294). Although not statistically significant, there was a trend of higher BARC ≥ 2 bleeding rates in LTPR patients (9/37 vs. 7/64, P = 0.076) (Suppl. Table S1). BARC ≥ 2 bleeding events were observed in 1 out of 9 (11.1%) patients on clopidogrel compared to 8 out of 28 (28.6%) on ticagrelor (P = 0.403). On the other hand, 5 bleeding events occurred in HTPR patients; 3 of them were BARC ≥ 2 (3/15 HTPR vs. 13/86 non-HTPR patients, P = 0.702) (Suppl. Table S1). Concerning antiplatelet regimens, among those who escalated to ticagrelor 1 out of 7 suffered from BARC ≥ 2 bleeding compared to 2 out of 8 patients who remained on clopidogrel (P = 1.000).

Regarding thrombotic complications, there were 4 patients who suffered an ischemic event under clopidogrel, 2 patients under ticagrelor and 1 patient with HTPR at discharge, under Rivaroxaban 15 mg monotherapy at 7th month.

## Discussion

During a transitional period for antithrombotic therapy from TAT to DAT, while incorporating NOAC instead of VKA, we have described the use of PFT in AF patients undergoing PCI, in the context of a real-life contemporary registry. In our study, PR and subsequent incidence of HTPR was greatly reduced in ticagrelor-treated patients compared to clopidogrel-treated patients. The observed HTPR incidence in clopidogrel-receiving anticoagulated patients is consistent with studies examining PR in patients on clopidogrel without anticoagulation [8, 9]. Among 103 patients on clopidogrel and rivaroxaban, HTPR and LTPR rates of 11.7% and 24.3% were described [10]. In contrast, the respective rates in our clopidogrel plus rivaroxaban-receiving patients were 32.4% and 14.7%; differences in populations and methodology used may have been implicated. Overall, observed PR levels were consistent with lack of effect of OACs in ADP-induced platelet reactivity measured by VerifyNow. Similar results have been reported in different clinical settings [15–17]. However, using other tests more sensitive to OAC effect might have unveiled differences. Moreover, PR decrease with ticagrelor was rather homogeneous, independently of anticoagulant administered. Of importance, our study provides for the first time, to our knowledge, data on PR under full-dose anticoagulation with rivaroxaban or dabigatran plus ticagrelor, in sizeable groups of patients. Data on dual pathway inhibition with the use of a either clopidogrel or ticagrelor in adjunct to a vascular dose of rivaroxaban (2.5 mg bid) have been recently reported in stable atherosclerotic heart disease patients; compared with DAPT, similar effects on platelet-mediated global thrombogenicity but reduced thrombin generation was demonstrated [18].

The potential clinical usefulness of PFT to guide individualized antithrombotic treatment in anticoagulated patients undergoing PCI is unclear [9, 13, 19]. In a randomized controlled trial involving Chinese patients and receiving warfarin and clopidogrel, PFT using vasodilator-stimulated phosphoprotein testing to up-titrate clopidogrel dosage in those with high platelet reaction index was applied. PFT-guided therapy resulted in decrease of MACCE, but no difference in survival or major bleeding events [20]. In our study, due to its observational nature, physicians’ behavior in response to patient’s PR result was variable; almost half of the patients remained on clopidogrel despite of HTPR demonstration. In addition, LTPR on ticagrelor practically had no impact on P2Y_12_ inhibitor selection. This likely reflects the well described very low PR achieved in the great majority of non-anticoagulated ticagrelor-treated patients and the possible lack of predictive value for subsequent bleeding events [21–23]. Although in one third of HTPR patients aspirin was added post PFT, overall rates of TAT or DAT were not affected by HTPR or LTPR status. Clinical equipoise among physicians regarding use of this information in the absence of definitive clinical trial data demonstrating the efficacy of this approach is hypothesized.

In our population PFT failed to effectively differentiate subsequent bleeding events, although there was a trend of lower PR in patients with BARC ≥ 2 bleeding events compared to those with minor or no events, as well as, a trend of higher BARC ≥ 2 bleeding rates in LTPR patients. Differences in age, index event and aspirin administration at discharge between groups, as well as selection bias, have probably affected bleeding outcomes.

The pivotal trials comparing TAT versus DAT did not include comprehensive PFT or genotyping in their design; thus, a recommendation for routine testing in this setting cannot be established and PFT clinical usefulness remains unproved. Nevertheless, a low PR under clopidogrel in an anticoagulated patient who is -by default- in high bleeding risk, might dictate omitting aspirin and DAT adoption. In contrary, detecting HTPR under clopidogrel, could point towards the adoption of a more intense antiplatelet regimen, via continuation of aspirin -in the form of TAT- or switching to ticagrelor. Considering the frequent occurrence of HTPR on clopidogrel, significant concerns have been raised that the suggested by the guidelines default strategy of DAT with NOAC and clopidogrel, leaves up to one-third of patients without adequate platelet inhibition [24]. Although randomized controlled trials definitively showed the superiority of DAT over TAT regarding bleeding events, they were underpowered for ischemic events. A higher risk of mainly stent-related ischemic events associated with DAT over TAT was described in a recent meta-analysis, although not confirmed in another [25, 26]. Escalation of antiplatelet therapy, even PFT-guided, might be considered in selected high-risk patients to avoid falling into the concept of sacrificing efficacy for safety [27].

PFT to identify LTPR and accordingly de-escalate antithrombotic therapy by choosing DAT to avoid bleeding may be more realistic, since bleeding side-effects are common in such patients. Nevertheless, the number of patients treated with TAT seems to be continuously decreasing as more clinicians adopt DAT at discharge following PCI. In similar clinical scenario, genotyping has been proposed as an independent factor in tailoring of antithrombotic therapy [10].

### Limitations

Nonrandomized, registry data, with lack of statistical adjustments for differences in compared group characteristics are presented. Moreover, due to the nature of the study, results are depicted in an observational, descriptive manner. PFT sub-study was led to premature discontinuation due to completion of the main study and slower recruitment in sub-study, pertinent to unavailability of PFT in participant centers. The small sample size renders the study underpowered for clinical events and hinders propensity score matching. Nevertheless, significant differences in prespecified primary endpoints were achieved. The results of this study are insufficient to demonstrate a clinical benefit of PFT in this group of patients, though are hypothesis generating. Apixaban was underrepresented in our study, given the fact that patients’ enrollment was initiated prior to publication of the Antithrombotic Therapy after Acute Coronary Syndrome or PCI in Atrial Fibrillation (AUGUSTUS) trial [5]. Moreover, edoxaban is currently not available, and prasugrel use is limited in this context, due to lack of adequate data. Finally, patients of GRAPE-AF were recruited during a transitional period regarding trends in antithrombotic therapy and important scientific data accruement. Current patterns of antithrombotic treatment in AF patients post-PCI may therefore differ following the clearly demonstrated superiority of DAT with clopidogrel over TAT regarding safety and the proposal of DAT as the default strategy after one week or post discharge with class I recommendation [28].

## Conclusion

In AF patients on OAC undergoing PCI, PFT applied in a real-life contemporary registry demonstrated the more potent platelet inhibition provided by ticagrelor and no impact of selected OAC on ADP-induced platelet reactivity measured by VerifyNow. PFT might represent a tool for tailoring of antithrombotic therapy although further and larger studies are required to elucidate its role. With the foregoing limitations, this study could be informative for the practicing clinician balancing between the ischemic and bleeding hazard in such patients.

## Supplementary Information

Below is the link to the electronic supplementary material.

Supplementary material 1 (DOCX 212.9 kb)
