# Supplementary material for: Platelet function testing in atrial fibrillation patients undergoing percutaneous coronary intervention
Source: J Thromb Thrombolysis. 2022 Nov 12;55(1):42–50. doi: 10.1007/s11239-022-02723-4 (PMC9925488; doi:10.1007/s11239-022-02723-4)
Supplement: Supplementary file 1 — Supplementary material 1 (DOCX 212.9 kb) [file 11239_2022_2723_MOESM1_ESM.docx]

**SUPPLEMENTARY INFORMATION**

| **Suppl. Table S1**: One year bleeding events rates by BARC type according to platelet reactivity status | | | | | | |
| --- | --- | --- | --- | --- | --- | --- |
| **Bleeding BARC** | **HTPR**  **(n = 15)** | **non-HTPR**  **(n = 86)** | **P** | **LTPR**  **(n = 37)** | **non-LTPR**  **(n = 64)** | **P** |
| Type 1 | 2 (13.3%) | 19 (22%) | 0.731 | 7 (18.9%) | 14 (21.8%) | 0.724 |
| Type 2 | 2 (13.3%) | 7 (8.1%) | 0.619 | 5 (13.5%) | 4 (6.2%) | 0.282 |
| Type 3a | 1 (6.6%) | 2 (2.3%) | 0.386 | 2 (5.4%) | 1 (1.5%) | 0.552 |
| Type 3b | 0 (0%) | 4 (4.6%) | 1.000 | 2 (5.4%) | 2 (3.1%) | 0.622 |
| Type ≥ 2 | 3 (20%) | 13 (15.1%) | 0.702 | 9 (24.3%) | 7 (10.9%) | 0.076 |
| Total | 5 (33.3%) | 32 (37.2%) | 1.000 | 16 (43.2%) | 21 (32.8%) | 0.294 |

BARC = Bleeding Academic Research Consortium, LTPR = Low on Treatment Platelet Reactivity, HTPR = High on Treatment Platelet Reactivity

**
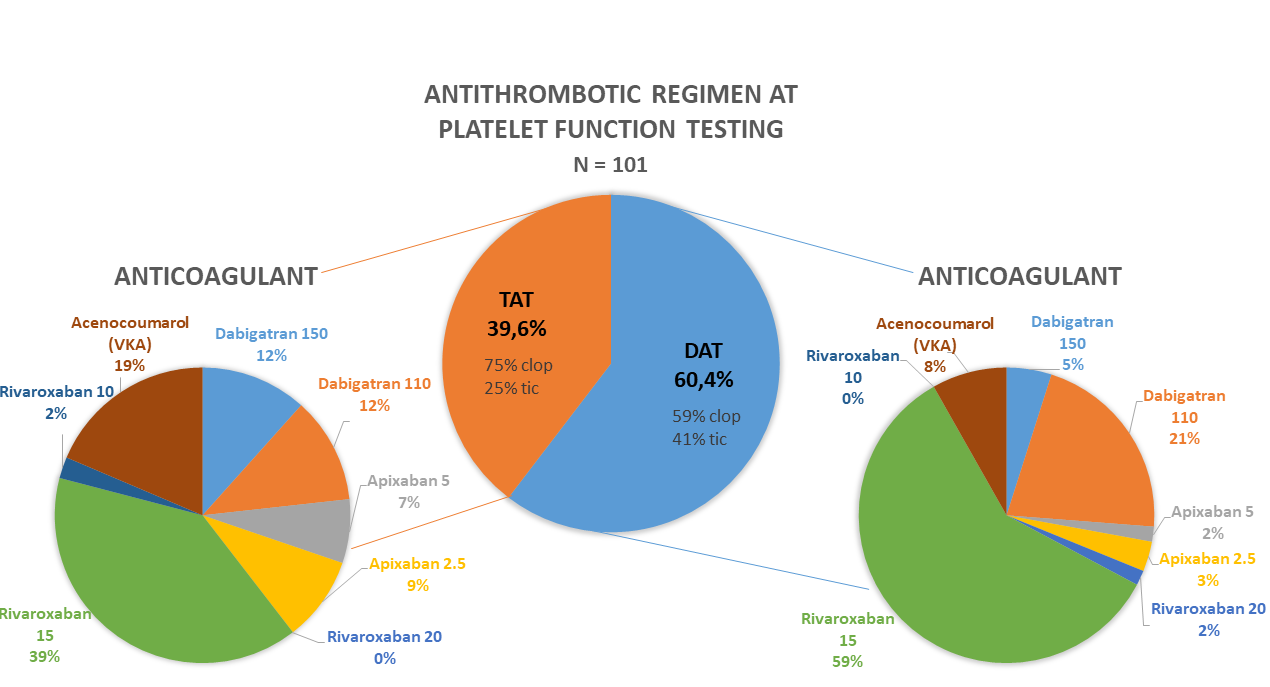
**

**Suppl. Fig. S1:** Antithrombotic regimen at discharge.

*DAT, double antithrombotic therapy; TAT, triple antithrombotic therapy; VKA, vitamin K antagonist*

#
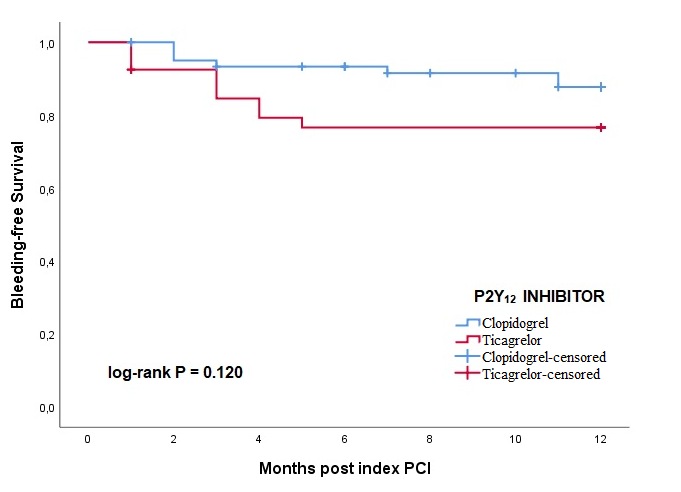
Suppl. Fig. S2: Kaplan–Meier survival curve for BARC≥2 bleeding-free survival during 12-month follow-up period.
